# Temperature variation in caves and its significance for subterranean ecosystems

**DOI:** 10.1038/s41598-023-48014-7

**Published:** 2023-11-25

**Authors:** Maria J. Medina, Dragan Antić, Paulo A. V. Borges, Špela Borko, Cene Fišer, Stein-Erik Lauritzen, Jose L. Martín, Pedro Oromí, Martina Pavlek, Ester Premate, Ken P. Puliafico, Alberto Sendra, Ana Sofia P. S. Reboleira

**Affiliations:** 1 Departamento de Biologia Animal, and Centre for Ecology, Evolution and Environmental Changes (cE3c) & CHANGE–Institute for Global Change and Sustainability, Faculdade de Ciências, Universidade de Lisboa, Campo Grande, 1749-016 Lisbon, Portugal; 2 Faculty of Biology, Institute of Zoology, University of Belgrade, Studentski Trg 16, 11 000 Belgrade, Serbia; 3 Department of Environmental Sciences and Engineering, Faculty of Agriculture and Environment, Centre for Ecology, Evolution and Environmental Changes (cE3c)/Azorean Biodiversity Group & CHANGE–Institute for Global Change and Sustainability, Universidade dos Açores, 9700-042 Angra do Heroísmo, Açores, Portugal; 4 Department of Biology, Biotechnical Faculty, SubBioLab, University of Ljubljana, Jamnikarjeva 101, 1000 Ljubljana, Slovenia; 5 Department of Earth Science, University of Bergen, Allegt. 41, 5007 Bergen, Norway; 6 Department of Biosciences, Centre for Ecological and Evolutionary Synthesis (CEES), University of Oslo, 0316 Oslo, Norway; 7 Parque Nacional del Teide, C/Dr. Sixto Perera González, 25. La Orotava, Tenerife, Spain; 8 Department of Animal Biology, University of La Laguna, Tenerife, Spain; 9 Ruđer Bošković Institute, Zagreb, Croatia; 10 Croatian Biospeleological Society, Zagreb, Croatia; 11 Center for Environmental Management of Military Lands, Colorado State University, Asan, Guam; 12 Colecciones Entomológicas Torres-Sala, Servei de Patrimoni Històric, Ajuntament de València, Passeig de La Petxina, 15, 46008 València, Spain; 13 Departament de Didàctica de Les Cièncias Experimentals I Socials, Facultat de Magisteri, Universitat de València, Avda. Tarongers 4, 46022 València, Spain; 14 Natural History Museum of Denmark, University of Copenhagen, Universitetsparken 15, 2100 Copenhagen, Denmark

**Keywords:** Ecology, Ecosystem ecology

## Abstract

Climate change affects all ecosystems, but subterranean ecosystems are repeatedly neglected from political and public agendas. Cave habitats are home to unknown and endangered species, with low trait variability and intrinsic vulnerability to recover from human-induced disturbances. We studied the annual variability and cyclicity of temperatures in caves vis-à-vis surface in different climatic areas. We hypothesize that cave temperatures follow the average temperature pattern at the surface for each location with a slight delay in the signal, but we found three different thermal patterns occurring in caves: (1) high positive correlation and a similar thermal pattern to the surface, (2) low correlation and a slight thermal delay of the signal from the surface, and (3) high negative correlation with an extreme delay from the surface. We found daily thermal cycles in some caves, which may potentially control the circadian rhythms of cave organisms. Our results show that caves had lower thermal amplitude than the surface, and that thermal averages within caves approximately correspond to the to the annual average of surface temperature. Caves buffer external temperature and act as refugia for biota in extreme climatic events. Likewise, temperature increases at surface will lead to increment in caves, threatening subterranean biota and ecosystem services.

## Introduction

Climate change is a main driver of biodiversity loss, affecting species’ geographical distribution, ecosystem functioning and services (human benefits provided by the ecosystems)^1–3^. These include support, provisioning, regulation and cultural services^4^. Thus, understanding how global warming affects all ecosystems is crucial^3^.

Our ability to make predictions regarding climate change on the surface has increased significantly over the last decades^2^. Nevertheless, below the ground hides a vast subterranean ecosystem, whose response to climate change remains largely unknown^5^. Caves are the most accessible underground ecosystems and constitute a window to study the vast dimension of the subterranean domain, that includes the mesocavernous shallow environment (MSS), a terrestrial shallow subterranean environment composed of the interstitial spaces between rocky fragments^6,7^. Caves lack light and are known to have stable environmental conditions, with surface influence decreasing with depth^5–11^. Temperature in caves show lower amplitude than at the surface^7,12^, although stable temperature in caves has been directly linked to average annual temperature at the surface for the same location^8,11–13^. Caves, with their isolated habitats and stable environmental conditions, serve as excellent models for predicting ecological responses to various environmental stressors, such as climate change^9,13–15^. In fact, a long-term thermal variation study over 13 years in an ice cave has found a warming trend on cave temperatures^16^.

Caves harbour unique organisms with specific adaptations to the peculiar conditions of subterranean habitats, including many short-range endemics, representing independent colonisations of surface ancestors, many ancient lineages and countless species yet to be discovered^5,17^. These ecosystems provide multiple benefits to humans^17,18^. They include the largest spaces for groundwater storage, but also water purification, in which subterranean biota plays the crucial role in the degradation of organic matter and pollutants^5,18,19^. In addition, caves are fundamental shelters for a variegated set of species, some of which are already known as being at risk of extinction, including bats^17^. Further, caves can provide opportunities for outdoor recreation, provide a unique environment for ecological end evolutionary scientific research, and have cultural and spiritual significance for indigenous communities and other groups^5,17^. Considering the importance of these ecosystems, understanding the thermal behaviour in caves is crucial to predict their fate under the ongoing global warming^5,13,17,20,21^.

We studied the temperature variation of deep zones in caves (those that do not receive any light from the surface and are more isolated) and on their respective surface (above the deep part of the cave), at sediment level for one year. Our hypothesis is that the temperature in the deep zones of caves follows the pattern of the average temperature at the surface for each location, expressing a slight delay and lower amplitude. We selected 12 caves located in different climatic zones, from tropical to cold continental climates, to study the relationship between surface and cave temperatures. We analysed the overall amplitudes, thermal patterns among seasons and months, and unveiled cyclicity patterns. Understanding how temperature in caves is related to surface temperatures in different climatic areas is crucial for predicting how climate change will affect subterranean ecosystems and consequently the ecological quality of groundwater.

## Results

### Temperature variation in caves vs on the surface

**Figure 1:**
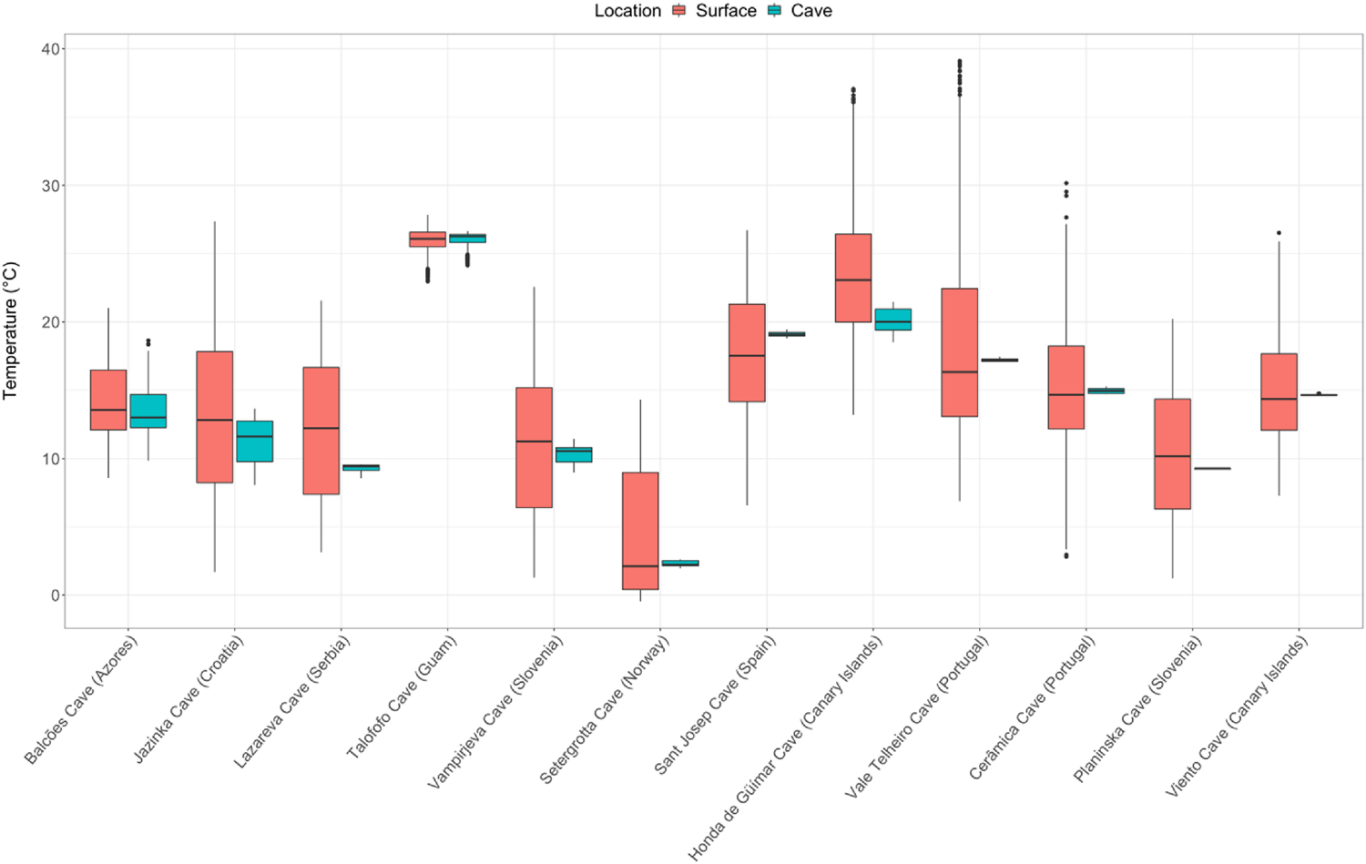
Thermal amplitude of deep zones of caves and their respective surface.

We found that temperature was consistently more stable in the caves than at the surface, with the lowest amplitudes occurring in the caves (Fig. 1, Table 1, Tables S2-S13). The highest temperature was recorded at the surface in southern Portugal (Vale Telheiro: 39.1 °C) and the highest cave temperature in Guam (Talofofo: 26.7 °C). In comparison, the lowest temperatures were recorded in northern Norway (− 0.5°C at the surface and 2 °C in the Setergrotta Cave) (Table 1). For this high latitude location, it should be noted that surface air temperatures may drop beyond − 25°C during winter (e.g. − 24.2 °C in February 2020, data from the “Norwegian Meteorological Institute”). The observed constancy of winter temperature in the other locations is due to up to 2 m thick snow cover at the surface site. We found a statistically significant difference between the cave and surface mean daily temperature (MDT) for seven of the studied locations (Balcões, Honda de Güímar, Jazinka, Cerâmica, Lazareva, Planinska and Sant Josep) (Table 1).

**Table 1:** Statistics and comparison between deep zones of caves (C) and their respective surfaces (S).

Location	Zone	Min T		MDT		AAT		Max T		TA		Wilcoxon (C and S)	Correlation	C SD (24 h)	S SD (24 h)
		C	S	C	S	C	S	C	S	C	S				
Balcões (Azores)	Csb	9.9	8.6	13.4	14.1	13.4	14.1	18.6	21.0	8.8	12.4	$p=0.004$	$r\left(P\right)=0.93$	0.022	4.5
													$$p<0.001$$		
Honda de Güímar (Canary Islands)	BWk	18.5	13.2	20.1	23.5	20.1	23.5	21.5	37.1	3.0	23.9	$p<0.001$	$r \left(P\right)=0.53$	0.301	801.1
													$$p=0.014$$		
Jazinka (Croatia)	Csa	8.1	1.7	11.4	13.1	11.4	13.1	13.6	27.4	5.6	25.7	$p<0.001$	$r \left(P\right)=0.85$	0.263	195.2
													$$p<0.001$$		
Lazareva (Serbia)	Cfa	8.6	3.1	9.3	12.0	9.3	12.0	9.6	21.6	1.0	18.4	$p<0.001$	$r\left(Sp*\right)=0.85$	0.0004	10.3
													$$p<0.001$$		
Talofofo (Guam)	Am	24.2	23.0	26.1	26.0	26.1	26	26.7	27.9	2.5	4.9	$p=0.476$	$r\left(P\right)=0.90$	0.065	12.8
													$$p<0.001$$		
Setergrotta (Norway)	Dfc	2.0	-0.5	2.3	4.4	2.3	4.4	2.6	14.3	0.6	14.7	$p=0.801$	$r\left(Sp\right)=0.77$	NDC	6.5
													$$p<0.001$$		
Vale Telheiro (Portugal)	Csa	17.1	6.9	17.2	18.0	17.2	18	17.4	39.1	0.4	32.2	$p=0.087$	$r\left(P\right)=-0.62$	NDC	410.9
													$$p<0.001$$		
Cerâmica (Portugal)	Csb	14.7	2.8	14.9	15.1	14.9	15	15.3	30.2	0.6	27.3	$p=0.018$	$r\left(Sp\right)=-0.87$	NDC	369.3
													$$p<0.001$$		
Planinska (Slovenia)	Cfb	9.2	1.2	9.3	10.3	9.3	10.3	9.3	20.2	0.1	19.0	$p=0.001$	$r\left(P\right)=-0.79$	NDC	39.0
													$$p<0.001$$		
Vampirjeva (Slovenia)	Dfb	9.0	1.3	10.4	10.9	10.4	10.9	11.4	22.6	2.4	21.3	$p=0.801$	$r\left(Sp\right)=0.32$	0.002	43.4
													$$p<0.001$$		
Sant Josep (Spain)	BSk	18.8	6.6	19.1	17.7	19.1	17.7	19.4	26.7	0.6	20.1	$p<0.001$	$r\left(Sp\right)=-0.21$	NDC	40.3
													$$p<0.001$$		
Viento (Canary Islands)	BWk	14.6	7.3	14.6	14.9	14.6	14.9	14.7	26.5	0.2	19.2	$p=0.257$	$r\left(Sp\right)=-0.75$	0.00003	95.8
													$$p<0.001$$		

Min T—Minimum temperature, MDT—Mean daily temperature, AAT—Average Annual Temperature, Max T—Maximum temperature, TA—Thermal amplitude, Wilcoxon—Wilcoxon Signed Rank Test, Correlation—Correlation coefficient (P—Pearson correlation, Sp—Spearman correlation) for the complete data, SD—Spectral density, NDC—No daily cycle.

Annual thermal amplitudes in caves ranged from 0.1 °C in Planinska Cave in Slovenia to 8.8 °C in Balcões Cave on the Atlantic Island of Terceira in the Azores archipelago (Fig. 1, Table 1). The lowest thermal amplitudes at the surface were recorded in an Am (Tropical—Monsoon) climate zone in Guam (2.5 °C in the cave and 4.9 °C at the surface), and in a Csb (Temperate—Dry summer—Warm summer) climate zone in the Azores (8.8 °C in the cave and 12.4 °C at the surface), with Guam having the most similar values between cave and surface thermal amplitudes. The highest thermal amplitude was recorded at the surface in the vertical of Vale Telheiro Cave, southern Portugal (32.2 °C).

### Annual temperature variation patterns in caves compared to the surface

We found three main correlation patterns between annual cave and surface temperature variation: (1) caves with high positive correlation values to surface temperature, having the same thermal signal (0–1 months difference) as the surface but with a smaller amplitude (Balcões, Jazinka, Lazareva, Talofofo), (2) caves with low correlation to surface temperature, and with a slight thermal delay of the signal from the surface (1–4 months) (Vampirjeva, Setergrotta, Sant Josep, Honda de Güímar), and (3) caves with high negative correlation with the surface temperature, showing an extreme delay from the surface (5–6 months) (Vale Telheiro, Cerâmica, Planinska, Viento) (Table 1).

**Figure 2:**
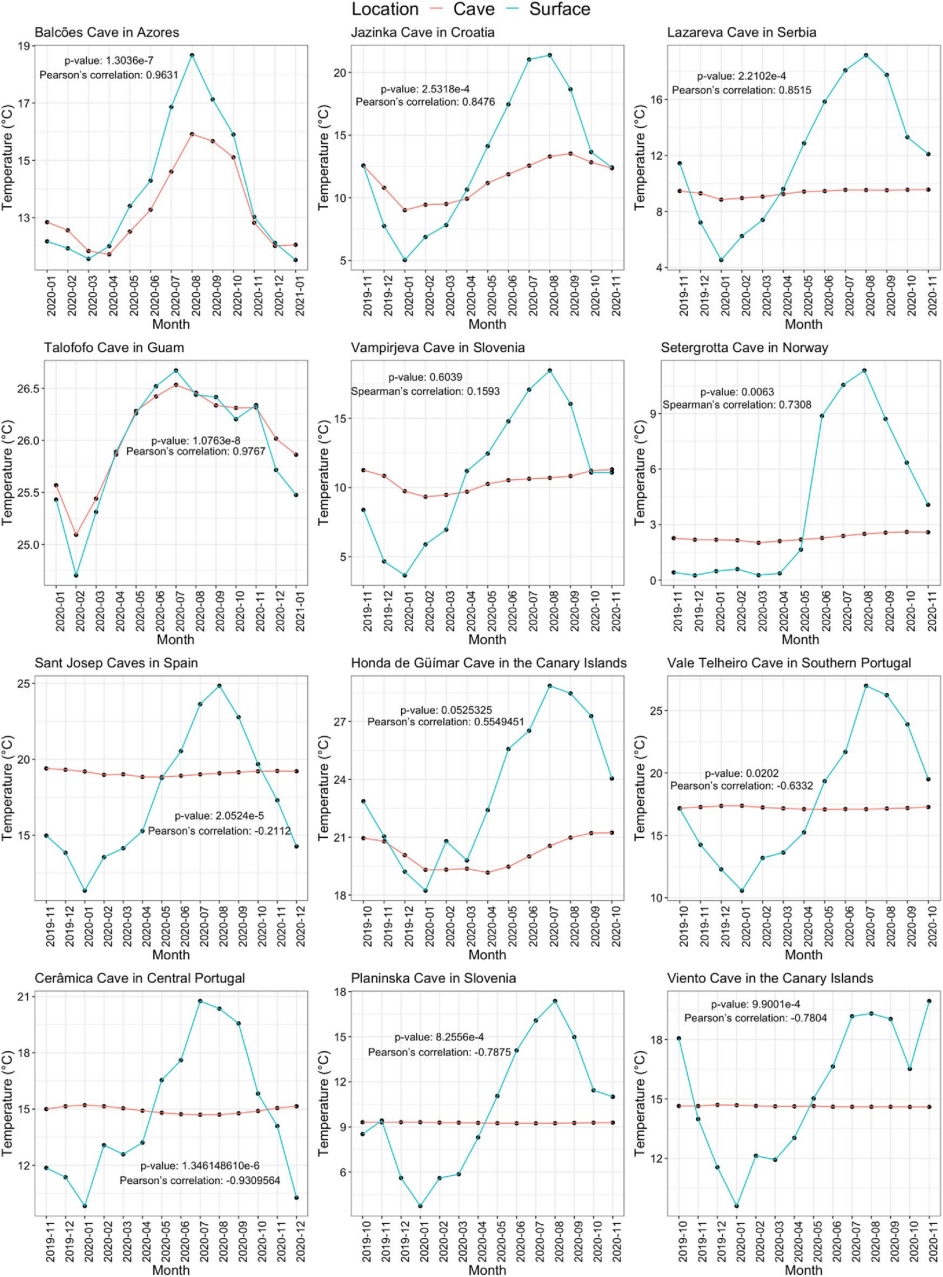
Average monthly thermal variation for the 12 studied locations along one year.

The caves with the lowest correlation with the surface (Vale Telheiro, Cerâmica, Planinska and Viento) show the most extreme delay in the coldest and warmest surface peaks (Fig. 2, Table 1). In these cases, the coldest temperatures inside the caves correspond to the warmest temperatures at the surface and vice versa. Contrarily, the caves that showed highest correlation with the surface show similar behaviour to the surface, with little to no delay.

### Thermal cyclicity in caves

**Figure 3:**
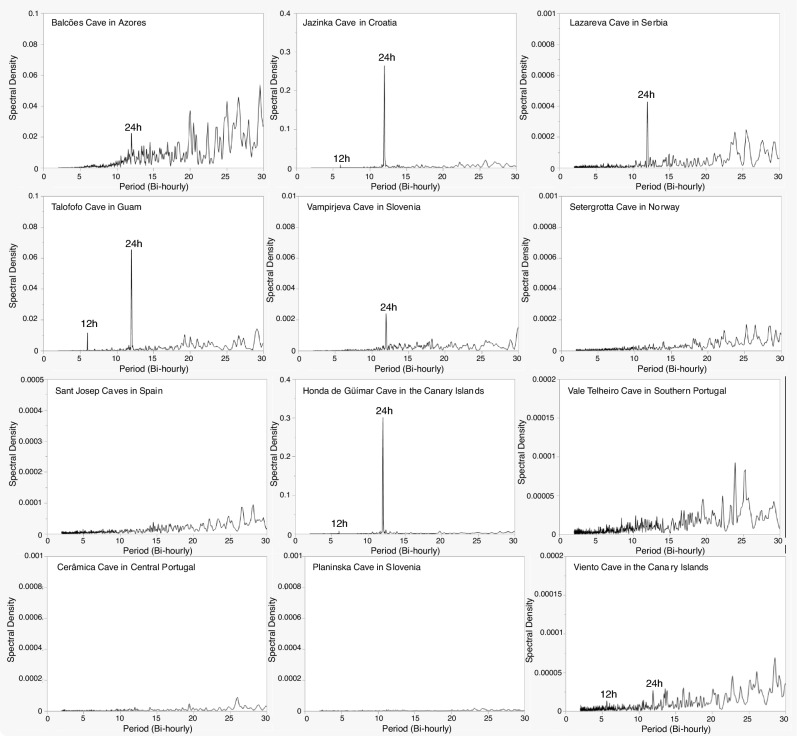
Spectral density analysis of temperature for deep zones of all studied caves.

We found daily thermal cycles in seven caves (Balcões in Azores, Jazinka in Croatia, Lazareva in Serbia, Talofofo in Guam, Vampirjeva in Slovenia, Viento and Honda de Güímar in the Canary Islands) (Fig. 3), with spectral density values always lower than those obtained for their surface (Table 1, Fig. 3, Figure S1). All other caves show no daily peaks, even at low spectral densities (Fig. 3). Caves with daily thermal cycles, show daily thermal cyclicity for individual seasons, but not necessarily for the four of them, while caves lacking daily thermal cyclicity, also lack cyclicity for seasonal data (Figures S2-S13).

## Discussion

### Temperature variation in caves vs. on the surface

The temperature in the caves was always more stable than on the surface. We found this across climate zones, lithologies, altitudes and latitudes and cave morphologies. The annual average temperature in caves is highly correlated with the average surface temperature. This agrees with other comparative studies that measured, simultaneously, temperature in caves and at their surface in tropical caves^8^, in cave air temperature in Slovenia^22^, and in cave-floor temperature in the Czech Republic^23^. This implies that temperature variations—such as those imposed by climate change—at surface will be reflected underground. Thermal stability in caves exercises strong selective pressure on all organisms thriving in the underground^13^, and the temperature increase due to climate change is known to threaten the ecological sustainability of subterranean ecosystems^5,20,21,24^.

### Annual temperature variation patterns in caves compared to surface

Cave temperatures are known to react “to long term temperature drifts with some delay”^18^, due to the inertia of the rock and fluids infiltration. Therefore, cave temperature is directly influenced by the temperature of the outer atmosphere^25^, and the surface heat transmission through the Earth’s upper crust is mainly via conductivity^26,27^. The signal delay has putatively been related to the depth of the cave zone^13^, i.e., in deep zones of caves forced ventilation seems to be the primary influence on temperature^27^.

Measuring temperature at soil level in the deep zone of each cave, we found three main correlation patterns in the annual temperature variation of the caves compared to the surface: (1) caves with high positive correlation with the surface temperature and identical thermal signal to the surface, but smaller amplitude, (2) caves with low correlation with the surface temperature and slight thermal delay of the signal from the surface, and (3) caves with high negative correlation to the surface temperature and extreme delay from the surface. This indicates that the thermal regime of the caves is influenced by the surface, but also by the individual characteristics of each cave. Rock properties, where igneous rocks have higher thermal conductivity than sedimentary rocks^26,28^, may lead to lower thermal delay in volcanic caves, which could explain the patterns found in Talofofo Cave (Guam) and Balcões Cave (Azores), but the opposite was found for Viento Cave (Tenerife), which was the most thermally stable of all caves measured. Cave morphology may affect the thermal regime as well, with deep parts of large caves usually being more stable^11,29^, which was observed for Planinska and Viento caves; however, Vale Telheiro (Portugal) is a very shallow cave (< 20m depth) and was also very stable. Latitude and altitude, which controls temperature at the surface and consequently in depth^26^. Moreover, air and water circulation contribute significantly to the regulation of cave temperatures^30–33^.

Caves are semi-closed complex systems and must therefore be understood as dynamic environments where the interaction of factors acting in the past plays a role in controlling the actual cave temperature. Air circulation is particularly relevant for caves with large and multiple entrances where air can be rapidly renewed, as in Guam, the Azores and Honda de Güímar Cave in Tenerife. Also, the geometry of cave passages may be a predictor of air circulation and consequently of temperature^34,35^. The surface air is denser in winter than in summer, resulting in cooler air entering the cave towards the lower points. The cold air pushes the warm air deeper into the most stable parts of the cave, forming an atmospheric “cul-de-sac”, where air renewal is more limited^36^, which might explain the pattern observed in caves with negative correlation to surface temperature^37^. These include some of the most biodiverse caves in the world^38,39^. On the other hand, the permanently undercooled caves, with often permanent presence of ice due to the downward gravitational movement of cold air (a so-called ice traps) are also known for high subterranean biodiversity, especially from the Dinarides^4^.

At higher latitudes and altitudes, seasonal ice layers formed during the cold season act as a thermal buffer, resulting in a lower thermal amplitude inside caves during winter, due to the lack of percolating water during that season^30^. This happened in Setergrotta Cave in northern Norway where we recorded a plateau in winter and early spring.

Other factors may be influencing the cave’s temperatures such as surface vegetation, by providing shadow^13^, humidity^7,40,41^, and potentially the geothermal gradient where temperature increases with depth^36,42^, although in karst regions this effect seems to be buffered by the advection of groundwater^31^.

### Thermal cyclicity in caves

Despite the general thermal stability in caves compared to the surface, caves with temperatures highly correlated with the surface showed daily thermal cycles. We expected this cycle to occur in caves that are most influenced by their respective surface, such as those with multiple entrances, where airflow plays an important role in controlling the cave microclimate. This pattern has already been observed in tropical caves, but limited to the shallowest parts of the cave^8^. We found daily cycles in the deep zones of caves located in different climatic areas, suggesting that daily temperature cycles in deep parts of caves may be frequent.

Circadian rhythms are intimately related to environmental cues such as light, and regulate different processes in organisms^43,44^. Previously, it was assumed that there were no daily variations in caves that could exert control over organisms^43^. However, the observed daily thermal cycles could play an important role to mark the circadian rhythms in cave-adapted organisms. Cave-adapted biodiversity is controlled by ecological, climatological, temporal and geological conditions^45^, but interestingly, we can observe that some of the most biodiverse caves (Planinska, Vale Telheiro and Cerâmica) have no daily cycles, suggesting that thermal stability could be a factor promoting high species richness below ground.

Our findings on thermal patterns and cyclicity in caves are particularly relevant for studying the impact of climate change in subterranean ecosystems and niche partitioning, but also for speleothems genesis^46^, and paleoclimatic reconstructions^47^, with potential implications on our capacity to interpret historical data from cave records. Further studies are needed to disentangle the role of the different drivers influencing cave microclimates.

We studied the variation at soil level in the deepest parts of different caves in many parts of the world, but thermal stratification may also occur in caves^11,29,33,40,48^. Further studies should include the effect of thermal stratification within individual caves, because the variation in temperature across the cave zones and between the floor and the roof of a gallery may have a major impact on speleogenesis, the formation and maintenance of ice in caves and, consequently in the creation of distinct ecological niches^30^.

Subterranean ecosystems harbors 95% of the world’s freshwater resources available for direct human consumption and the largest water reservoir for plants and agriculture^5,46^. Consequently, it is pivotal to understand the factors that influence cave temperatures and how they may affect cave species and ecosystem services^18,20,21,24,46^. The Intergovernmental Panel on Climate Change (IPCC) synthesis report from 2014 confirms that climate change has and will continue to impact ecosystems and geographical species’ distribution at surface, which will be reflected underground, by mean annual surface temperatures increase but also by the decrease of rainfall and extreme climatic events^2^. While epigean/surface dwelling species may have the ability to disperse to other altitudes and latitudes, cave-adapted communities are isolated in caves and with none or very limited survival capacity at surface^49^. This is even more evident in terrestrial communities that may be doomed without the ability to disperse^50^. We observed that the average temperature in the deep zones of the caves reflects the average surface temperature for each cave, therefore, we expect the increase in surface temperature to be reflected in the underground. In addition to global climate change, other human activities are also known to increase temperature in subterranean ecosystems, such as proximity to cities^51–53^. Moreover, in caves where the temperature is highly dependent on the surface, such as Balcões in the Azores and Talofofo in Guam, climate extremes may even be detected inside the cave. However, this work also shows how difficult it is to explain the factors that control cave climate. This is still very understudied and should be explored further to better understand the degree to which caves depend on the surface, and how vulnerable they are to anthropogenic threats such as climate change.

## Methods

### Data collecting

**Figure 4:**
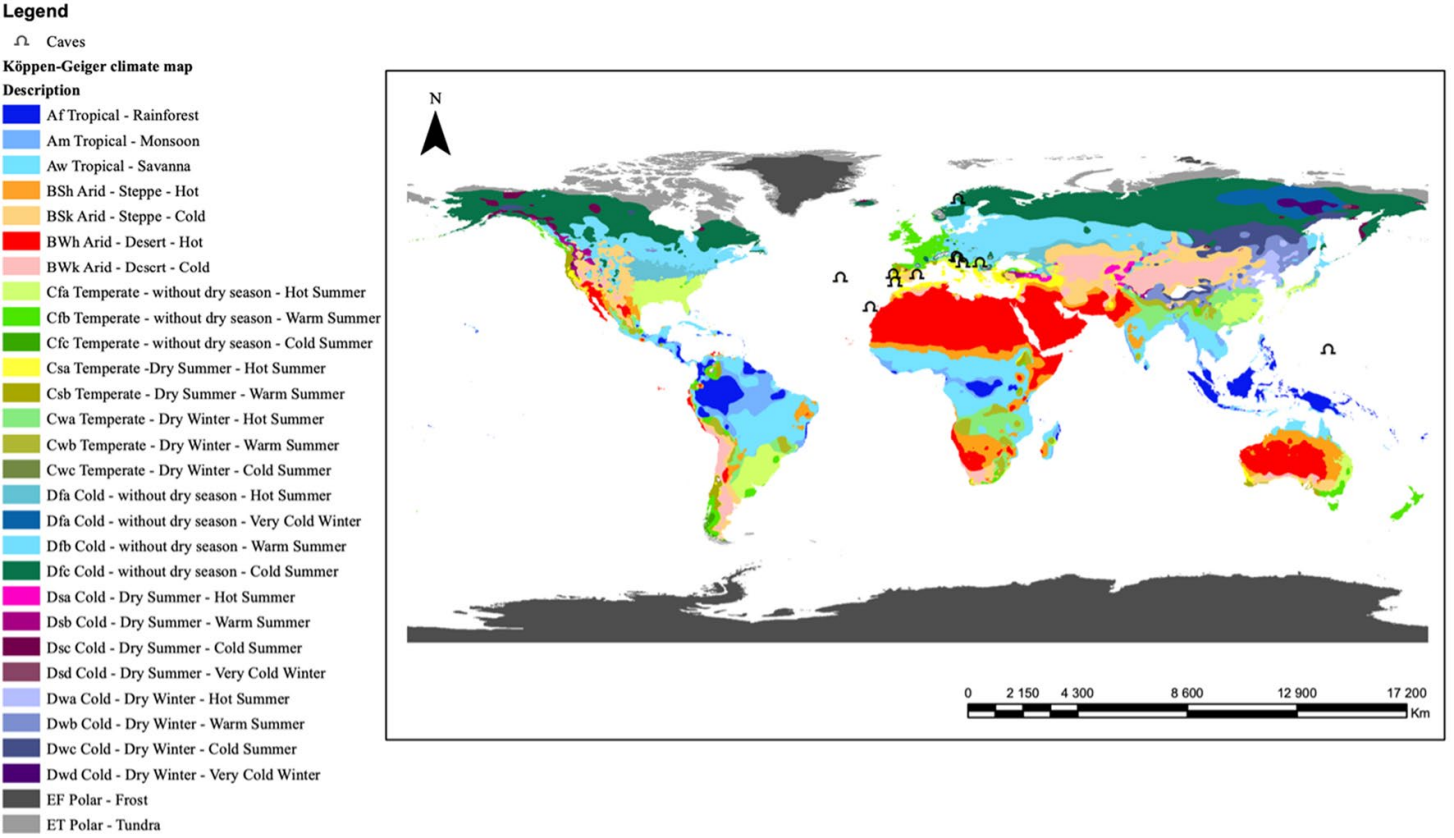
Locations of the studied caves across biomes. Map produced in ArcGIS (v10.7.1), with Köppen-Geiger climate classification layer adopted from Peel et al. ^54^.

We selected twelve caves in different climate zones: Tropical (Am Tropical—Monsoon), Arid (BWk—Arid—Desert—Cold, BSk—Arid—Steppe—Cold), Temperate (Cfa—Temperate—No dry season—Hot summer, Cfb—Temperate—No dry season—Warm summer, Csa—Temperate—Dry summer—Hot summer, Csb—Temperate—Dry summer—Warm summer), and Continental (Dfb—Continental—No dry season—Warm summer, Dfc—Continental—No dry season—Cold summer) (Fig. 4, Cave coordinates in Supplementary Table 1). For each location, temperature was recorded inside the cave in the deep zone to ensure minimal outside influence, and vertically above the cave at surface. Temperature data loggers (HOBO TidbiT v2) recorded temperature every two hours during a 12-month period, with an accuracy of ± 0.21 °C and a resolution of 0.02 °C. These were installed 2 cm into the soil at the surface and 2 cm into the sediment in the deepest part of each cave. The caves differ in terms of lithology, formation, size, climate zones, number of entrances, depth, and altitude. The map of localities was produced in ArcGIS (v10.7.1, https://www.esri.com/en-us/arcgis/products/arcgis-pro), over a Köppen-Geiger climate classification layer adopted from Peel et al. ^54^.

### Data analysis

Data was analysed with basic statistics in R and R Studio (v1.3.1073)^55^ and visualized with the package ggplot2^56^. Mean daily temperature (MDT), average annual temperature (AAT) and maximum and minimum temperatures were calculated for both the cave and the surface for each location. The correlation between the cave and surface environments for the annual and monthly data was analysed using a Pearson coefficient for variables with a linear relationship, while a Spearman coefficient was used for non-linear ones. To test whether the difference in MDT between each cave and respective surface pair was statistically significant, initially, a Shapiro–Wilk test was performed to check the normality of the variables. If the variables were parametric, we performed a t-test. If not, a two tailed Wilcoxon signed-rank test was used.

Thermal cyclicity was studied using spectral density analysis in JMP software (v16.0.0) to investigate the existence of 24 h cycles in caves. Spectral density analysis is used to study a signal’s periodicity linked to a cyclic behaviour^57^. This was performed searching for cyclical patterns in temperature data that repeats approximately every 60 h period. For this, the thermal cycles were analysed for the annual data of cave and surface for each location, and for the seasonal data, i.e. analysing independently the data from each season. Because the temperature was recorded every 2 h, the 24 h cycles are detectable as 12-time intervals, and the 12h cycles are detectable as 6-time intervals.

### Supplementary Information

Supplementary Figures.

Supplementary Tables.

## Data Availability

All data is available in supplementary material.
