# Supplementary material for: Temperature variation in caves and its significance for subterranean ecosystems
Source: Sci Rep. 2023 Nov 25;13:20735. doi: 10.1038/s41598-023-48014-7 (PMC10676404; doi:10.1038/s41598-023-48014-7)
Supplement: Supplementary file 1 — Supplementary Figures. [file 41598_2023_48014_MOESM1_ESM.docx]

**Temperature variation in caves and its significance for subterranean ecosystems**

Medina^1^, M.J., Antić, D.Ž. ^2^, Borges, P.A.V.^3^, Borko, Š.^4^, Fišer, C. ^4^, Lauritzen, S.E.^5,6^, Martín, J.L.^7^, Oromí, P.^8^, Pavlek, M.^9,10^, Premate, E.^4^, Puliafico, K.P.^11^, Sendra, A.^12,13^ & Reboleira, A.S.P.S.^1,14*^

^1^ Departamento de Biologia Animal, and Centre for Ecology, Evolution and Environmental Changes (cE3c) & CHANGE – Institute for Global Change and Sustainability, Faculdade de Ciências, Universidade de Lisboa, Campo Grande, 1749-016, Lisbon, Portugal

^2^ University of Belgrade, Faculty of Biology, Institute of Zoology, Studentski Trg 16, 11 000 Belgrade, Serbia

^3^ Centre for Ecology, Evolution and Environmental Changes (cE3c)/Azorean Biodiversity Group & CHANGE – Institute for Global Change and Sustainability, Faculty of Agriculture and Environment, Department of Environmental Sciences and Engineering, Universidade dos Açores, 9700-042 Angra do Heroísmo, Açores, Portugal

^4^ University of Ljubljana, Biotechnical Faculty, Department of Biology, SubBioLab, Jamnikarjeva 101, 1000 Ljubljana

^5^ Department of Earth Science, University of Bergen, Allegt. 41, 5007, Bergen, Norway

^6^ Department of Biosciences, Centre for Ecological and Evolutionary Synthesis (CEES), University of Oslo, 0316, Oslo, Norway

^7^ Parque Nacional del Teide, C/Dr. Sixto Perera González, 25. La Orotava, Tenerife, Spain

^8^ Dept. of Animal Biology, University of La Laguna, Tenerife, Spain

^9^ Ruđer Bošković Institute, Zagreb, Croatia

^10^ Croatian Biospeleological Society, Zagreb, Croatia

^11^ Center for Environmental Management of Military Lands, Colorado State University, Asan, Guam

^12^ Colecciones Entomológicas Torres-Sala, Servei de Patrimoni Històric, Ajuntament de València, Passeig de la Petxina, 15, 46008 València, Spain

^13^ Departament de Didàctica de les Cièncias Experimentals i Socials, Facultat de Magisteri, Universitat de València, Avda. Tarongers 4, 46022 València, Spain

^14^ Natural History Museum of Denmark, University of Copenhagen, Universitetsparken 15, 2100 Copenhagen, Denmark

* Corresponding author: asreboleira@fc.ul.pt

**SUPPLEMENTARY TABLES**

Table S1: Location and coordinates of the 12 studied caves (in excel).

Table S2: Raw data from the Balcões Cave and respective surface in Azores (in excel).

Table S3: Raw data from the Jazinka Cave and respective surface in Croatia (in excel).

Table S4: Raw data from the Lazareva Cave and respective surface in Serbia (in excel).

Table S5: Raw data from the Talofofo Cave and respective surface in Guam (in excel).

Table S6: Raw data from the Vampirjeva Cave and respective surface in Slovenia (in excel).

Table S7: Raw data from the Setergrotta Cave and respective surface in Norway (in excel).

Table S8: Raw data from the Sant Josep Cave and respective surface in Spain (in excel).

Table S9: Raw data from the Honda de Güimar Cave and respective surface in the Canary Islands (in excel).

Table S10: Raw data from the Vale Telheiro Cave and respective surface in Southern Portugal (in excel).

Table S11: Raw data from the Cerâmica Cave and respective surface in Central Portugal (in excel).

Table S12: Raw data from the Planinska Cave and respective surface in Slovenia (in excel).

Table S13: Raw data from the Viento Cave and respective surface in the Canary Islands (in excel).

**Supplementary figures**


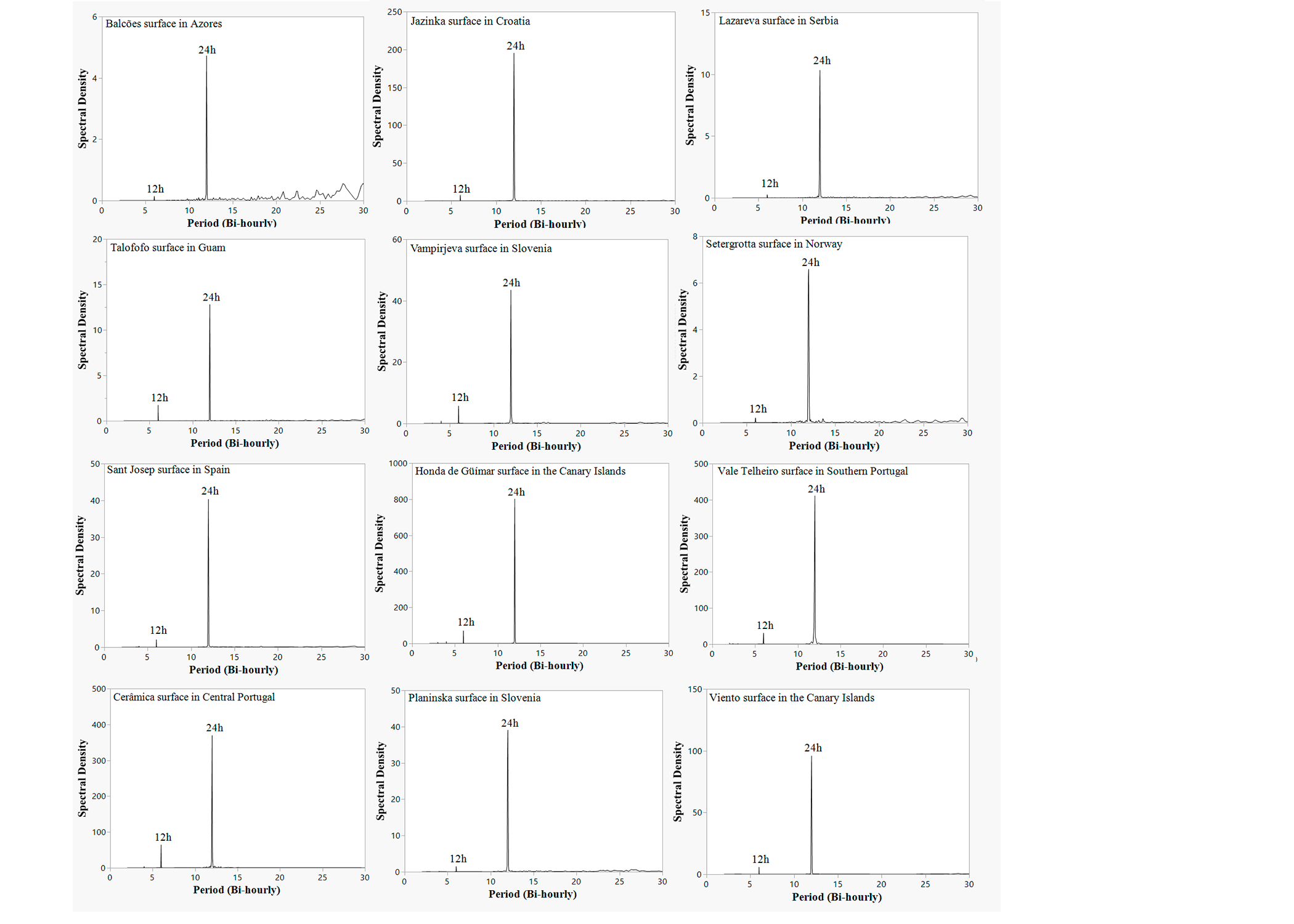


Figure S1: Spectral density analysis of temperature for deep zones of all studied caves respective surfaces.


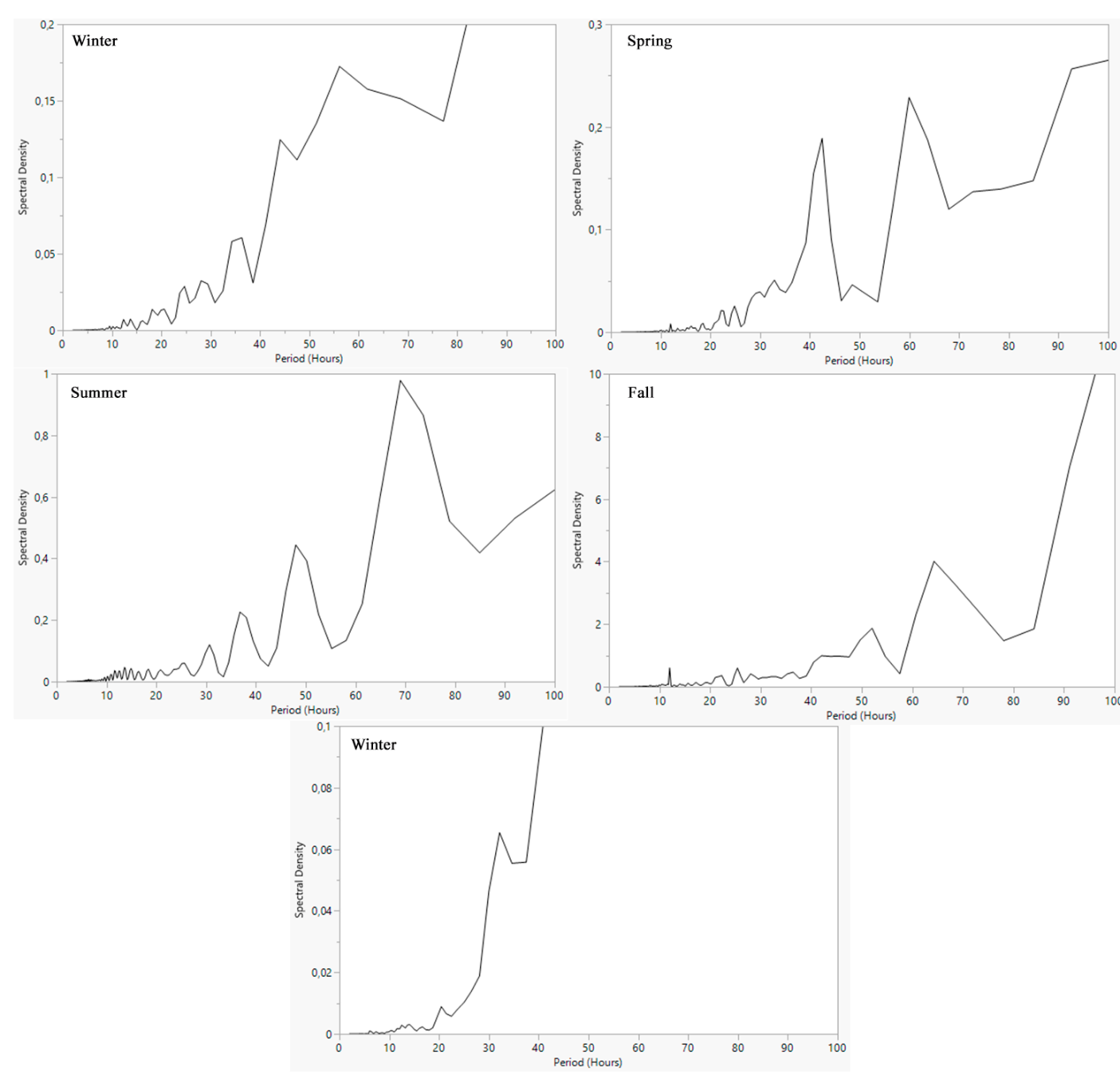


Figure S2: Seasonal spectral density analysis of temperature for the deep zone of the Balcões Cave (Azores).


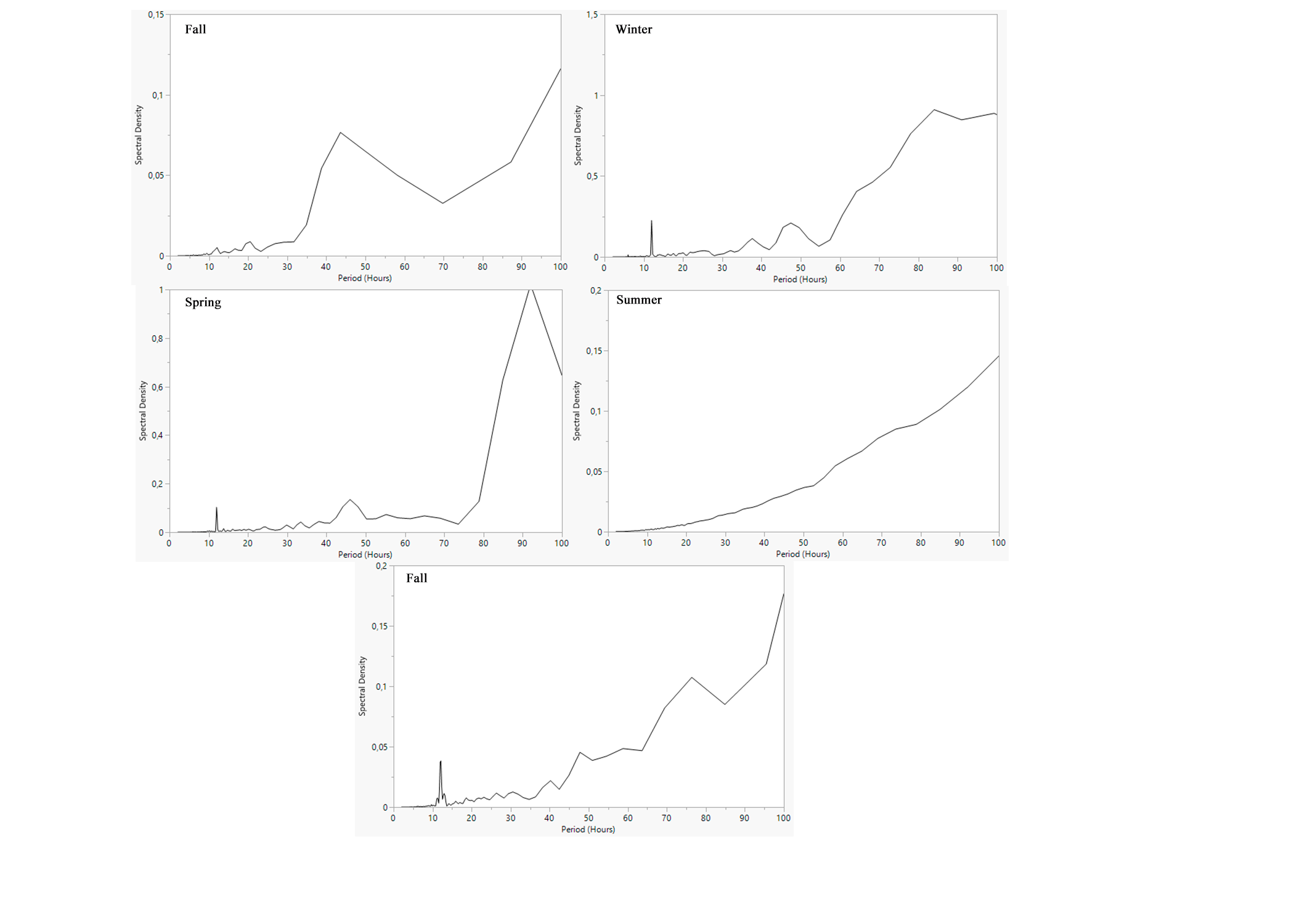


Figure S3: Seasonal spectral density analysis of temperature for the deep zone of the Jazinka Cave (Croatia).


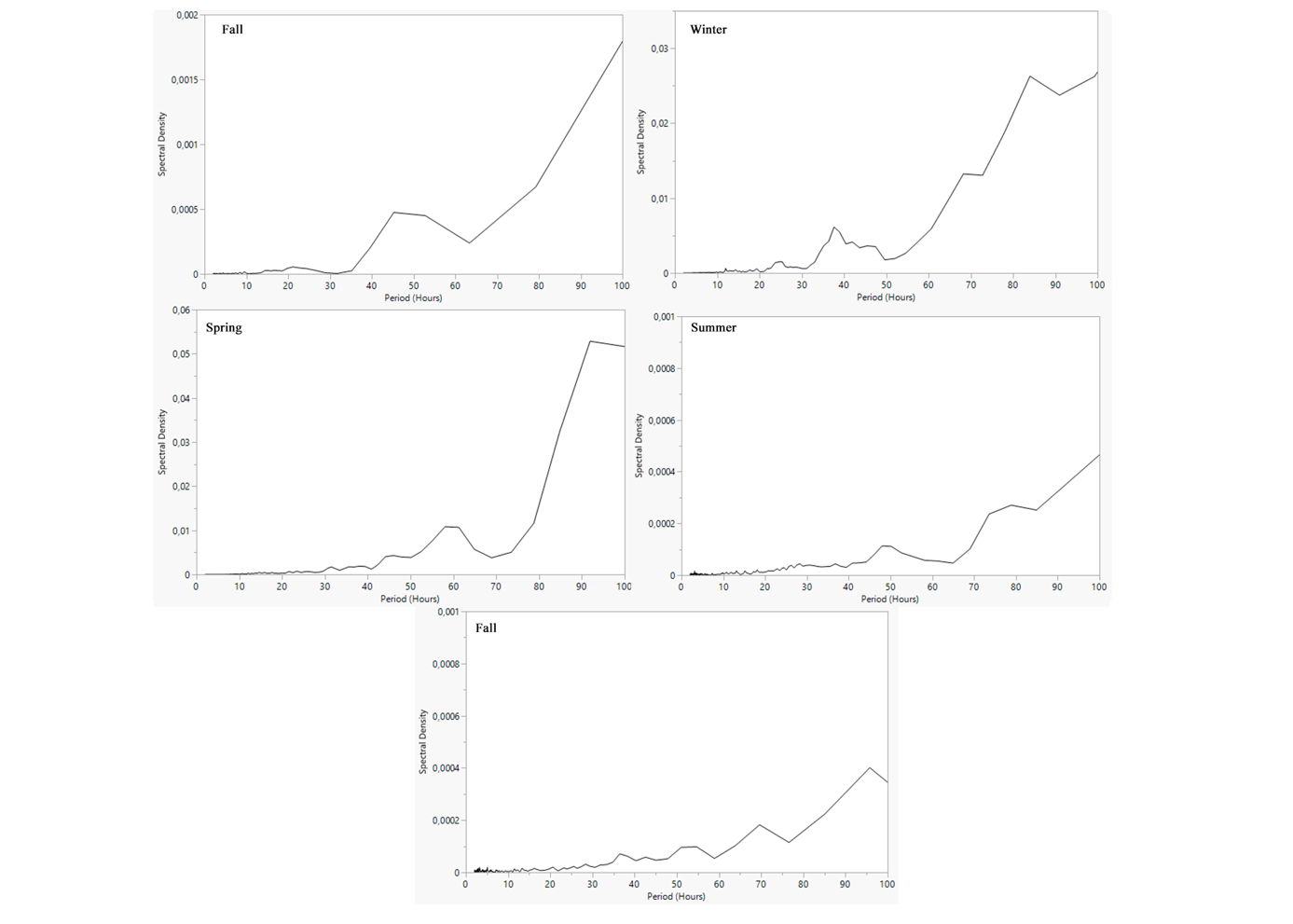


Figure S4: Seasonal spectral density analysis of temperature for the deep zone of the Lazareva Cave (Serbia).


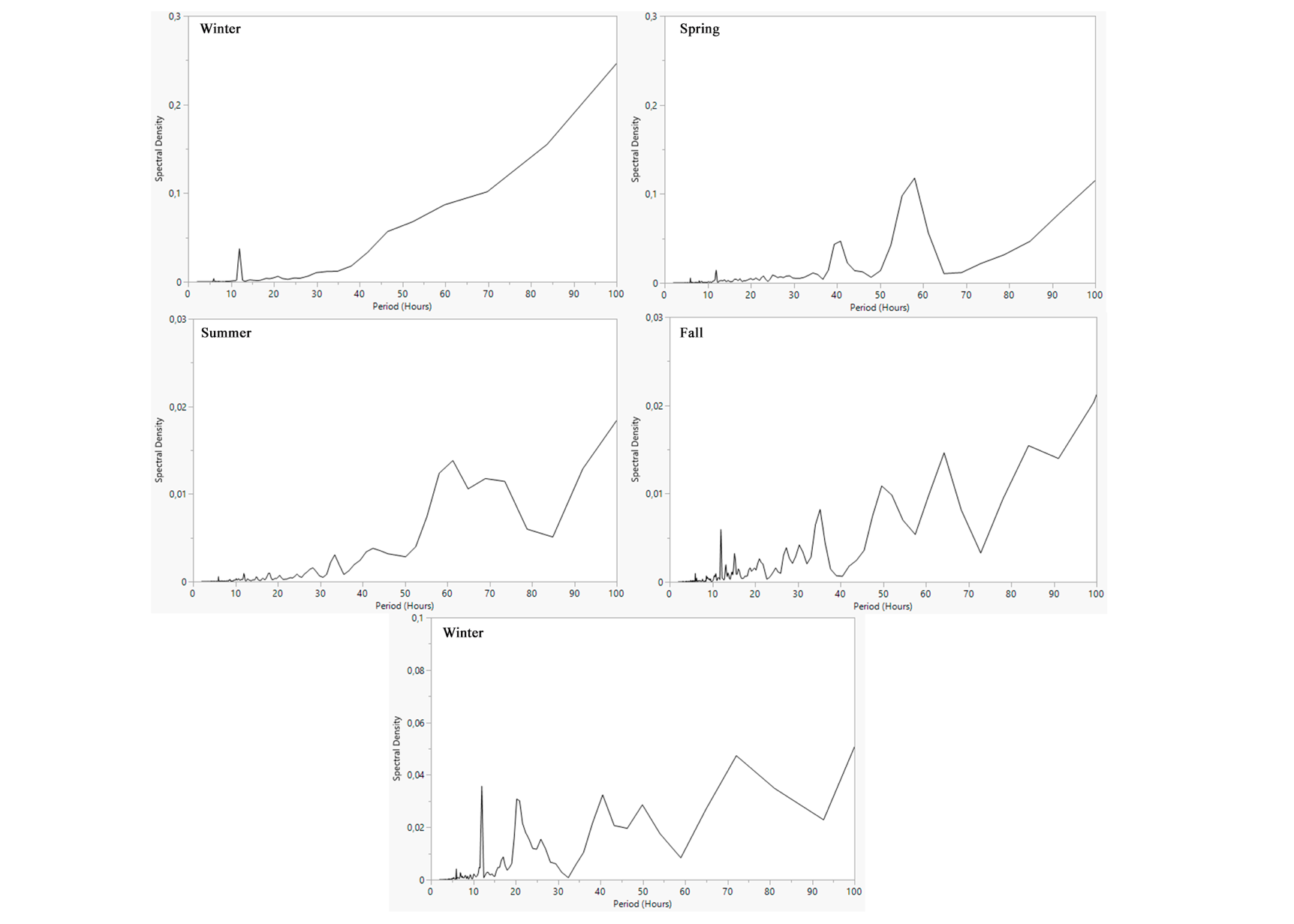


Figure S5: Seasonal spectral density analysis of temperature for the deep zone of the Talofofo Cave (Guam).


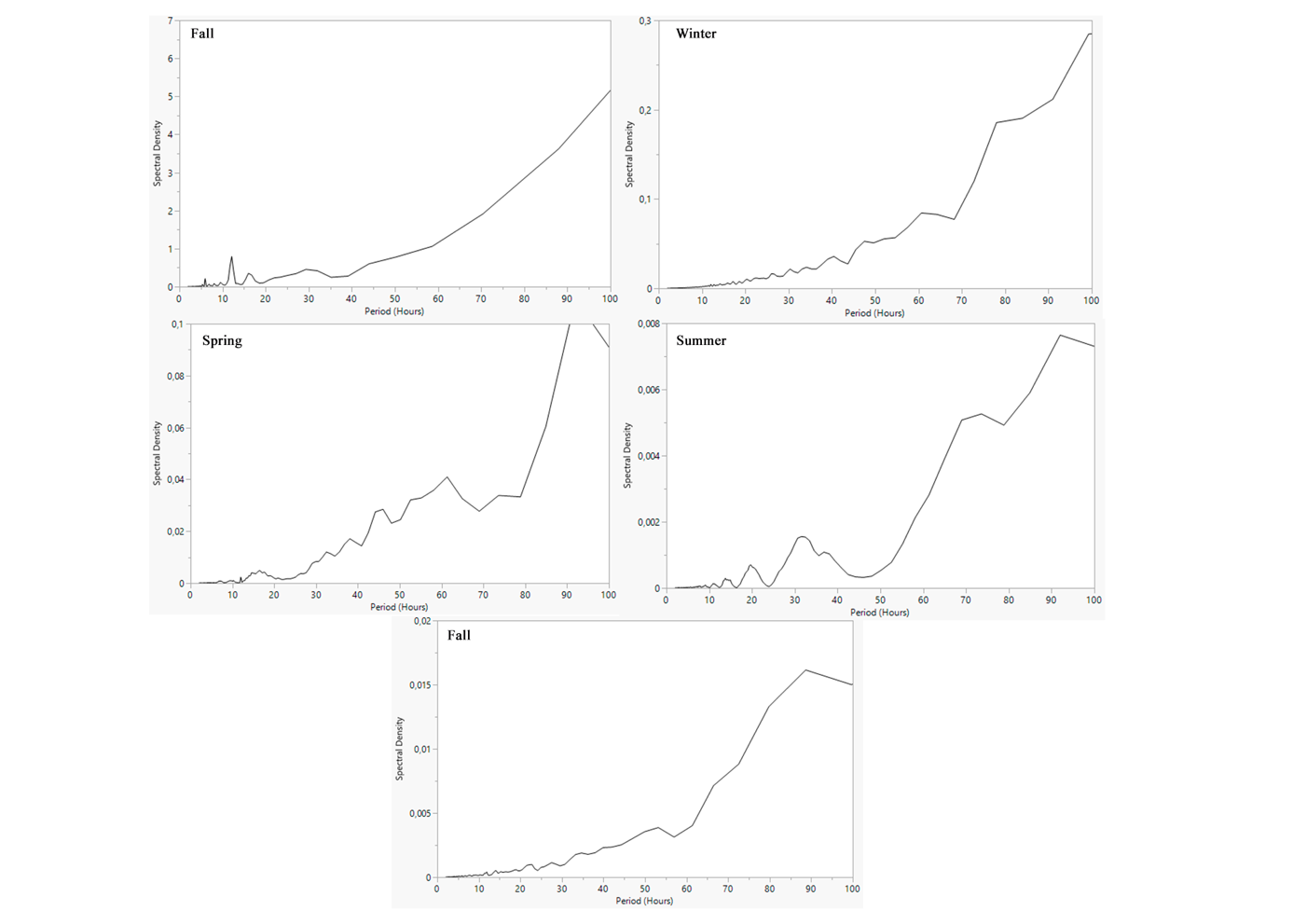


Figure S6: Seasonal spectral density analysis of temperature for the deep zone of the Vampirjeva Cave (Slovenia).


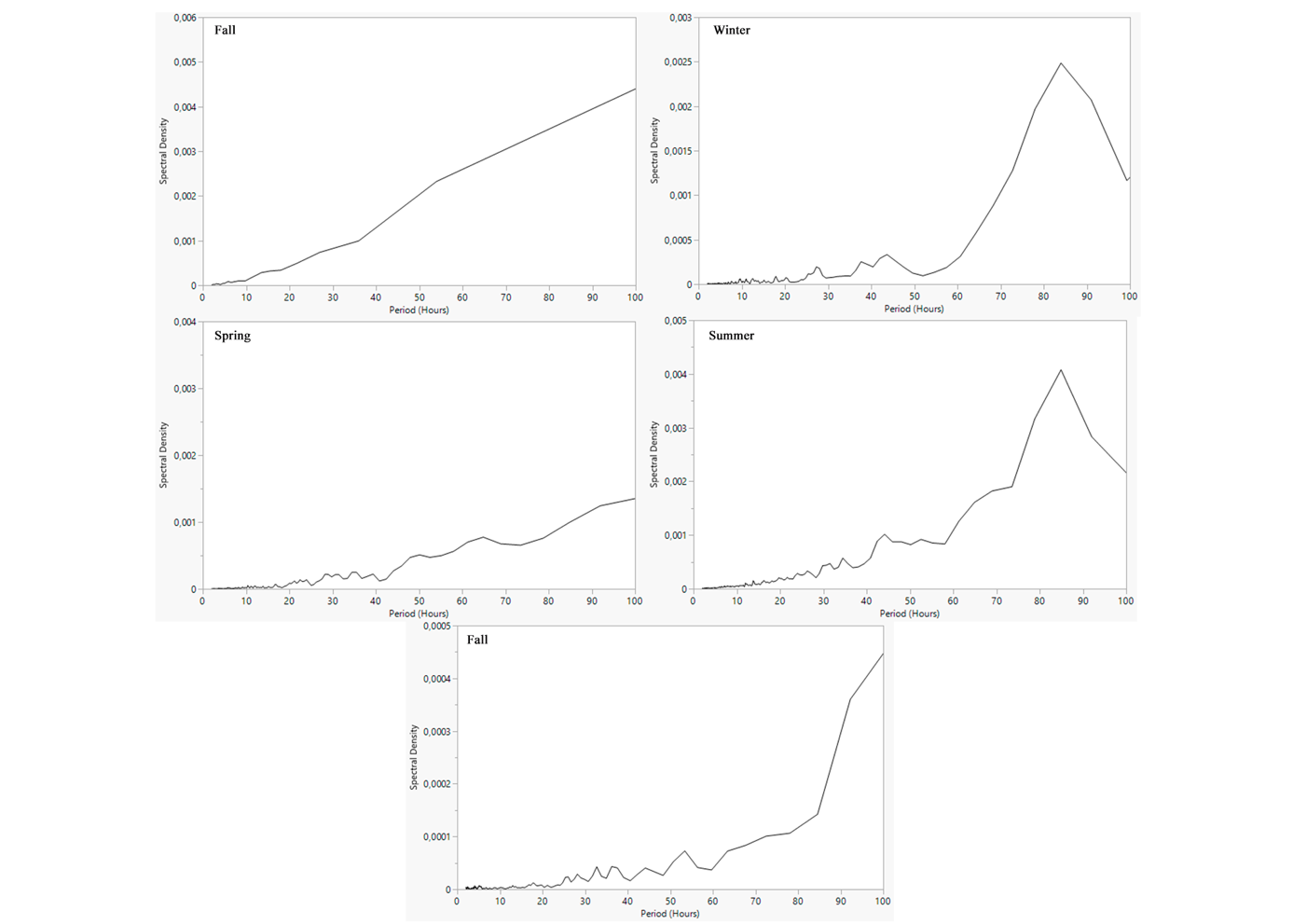


Figure S7: Seasonal spectral density analysis of temperature for the deep zone of the Setergrotta Cave (Norway).


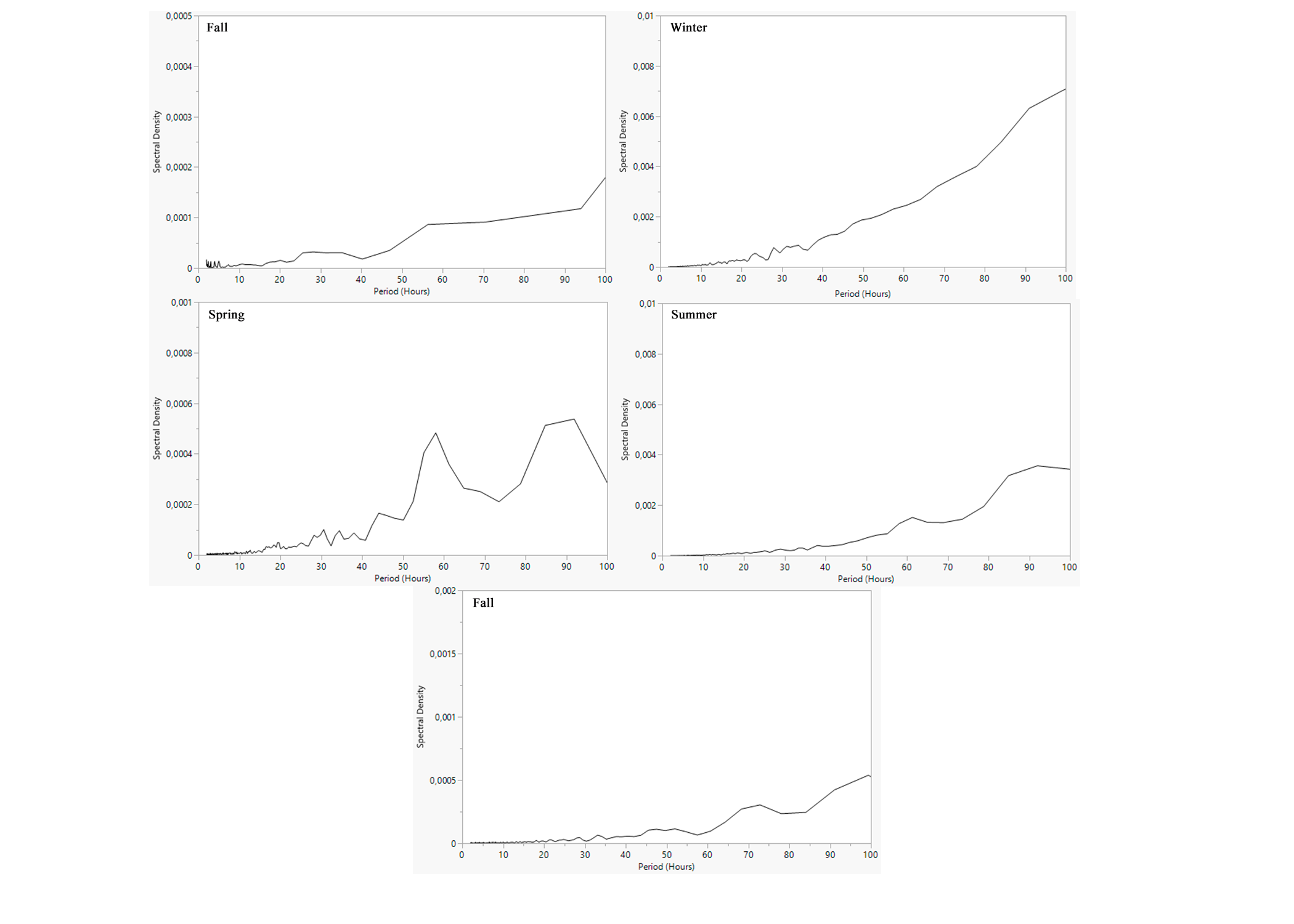


Figure S8: Seasonal spectral density analysis of temperature for the deep zone of the Sant Josep Cave (Spain).


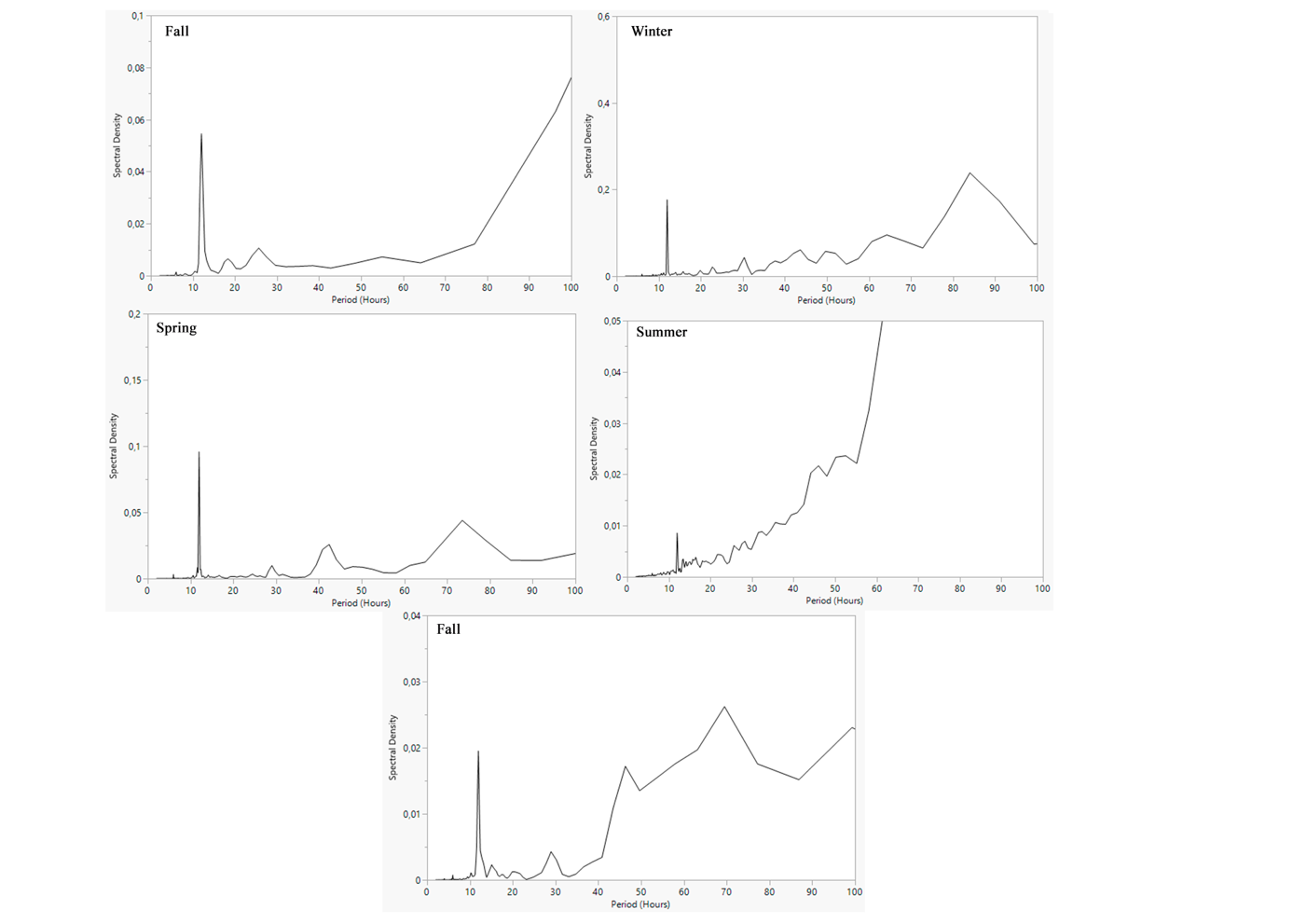


Figure S9: Seasonal spectral density analysis of temperature for the deep zone of the Honda de Güimar Cave (Canary Islands).


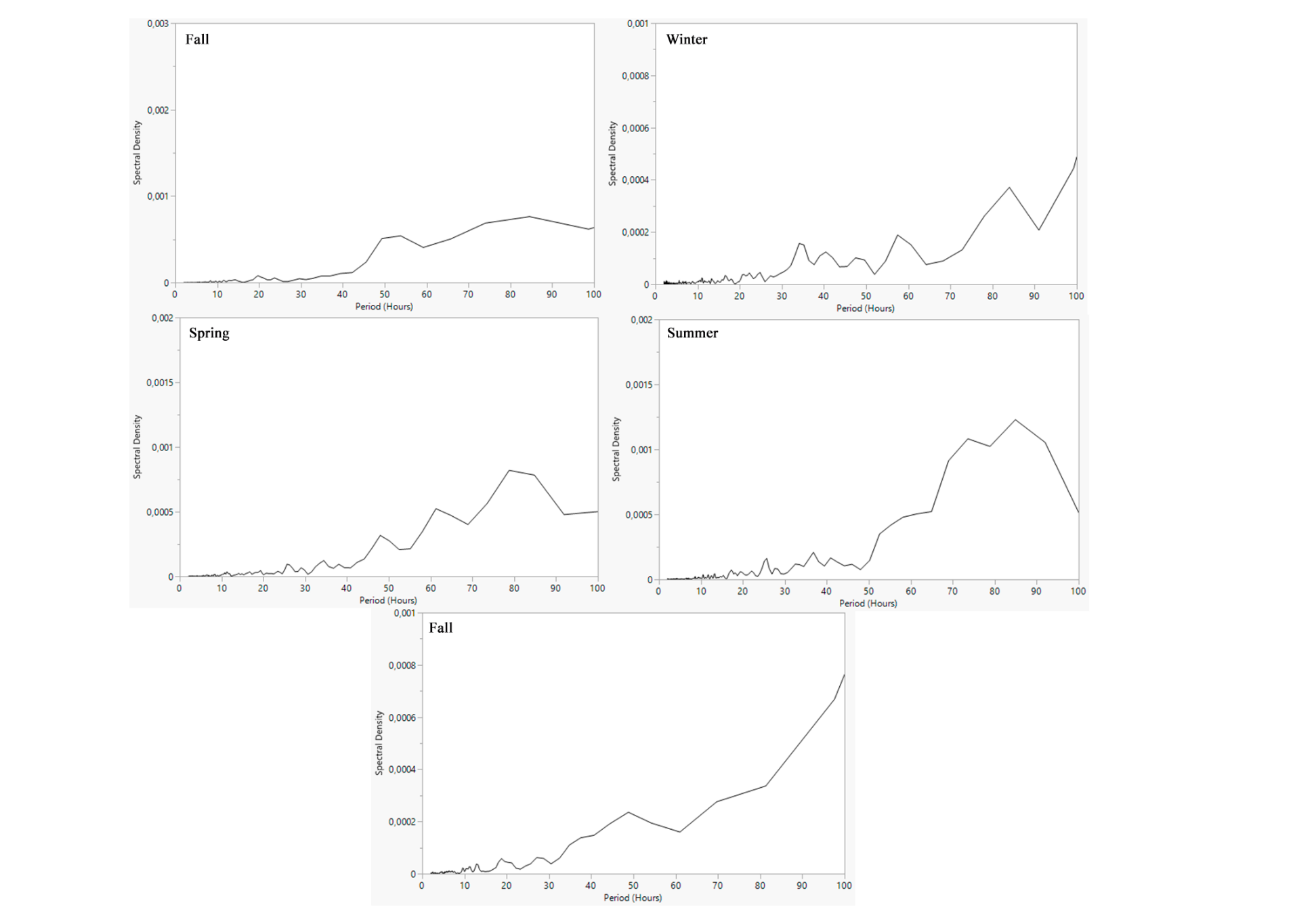


Figure S10: Seasonal spectral density analysis of temperature for the deep zone of the Vale Telheiro Cave (Southern Portugal).


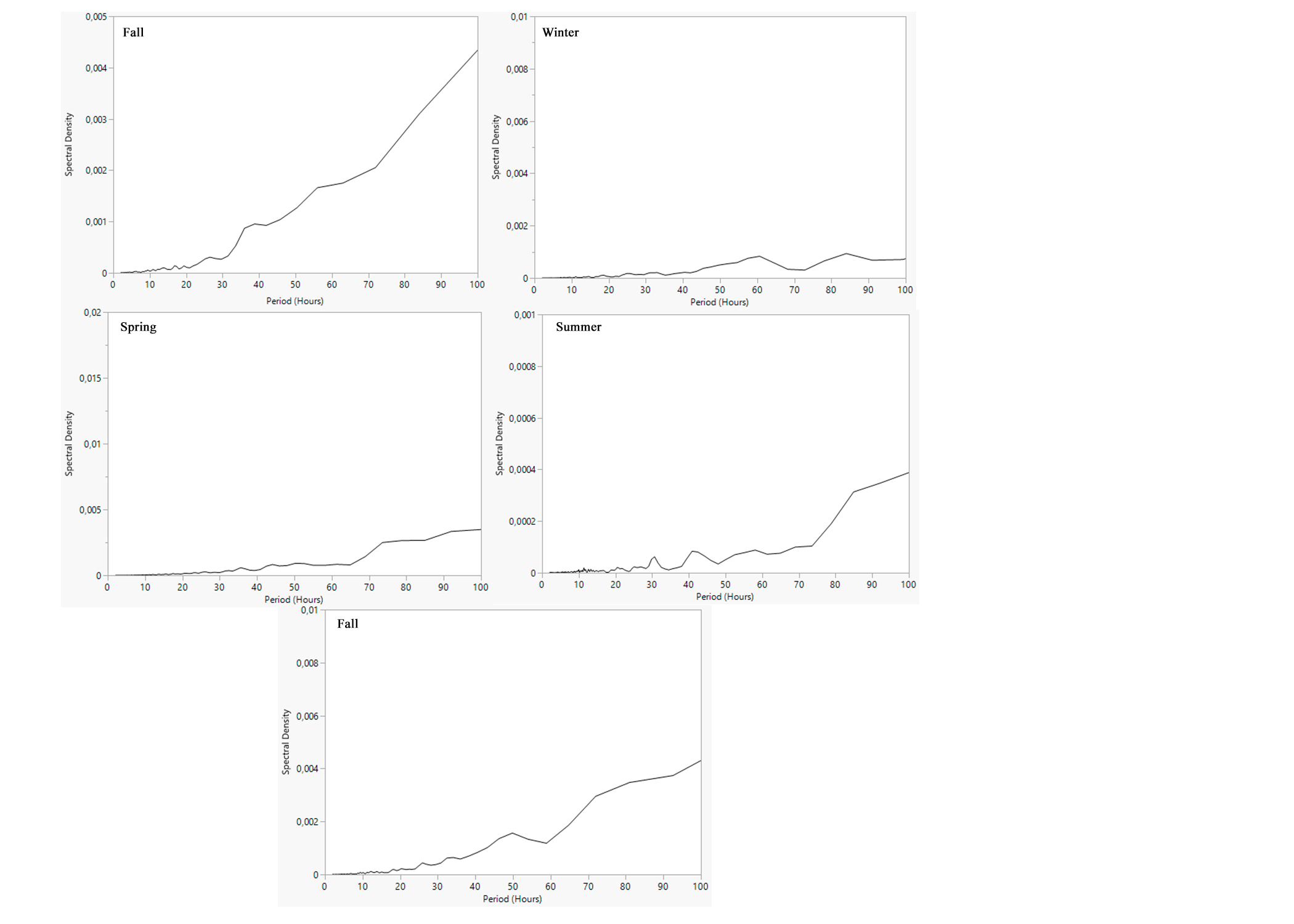


Figure S11: Seasonal spectral density analysis of temperature for the deep zone of the Cerâmica Cave (Central Portugal).


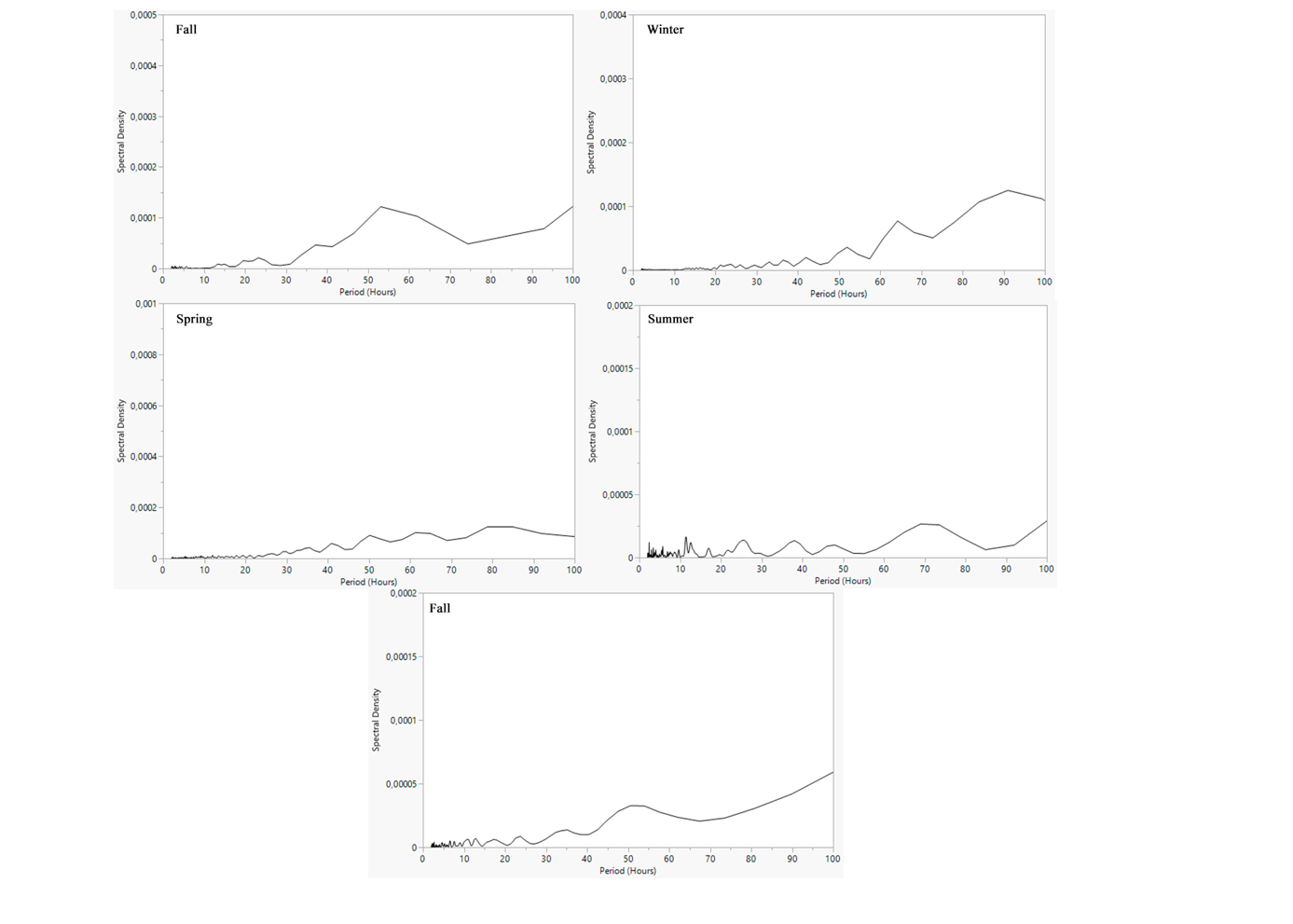


Figure S12: Seasonal spectral density analysis of temperature for the deep zone of the Planinska Cave (Slovenia).


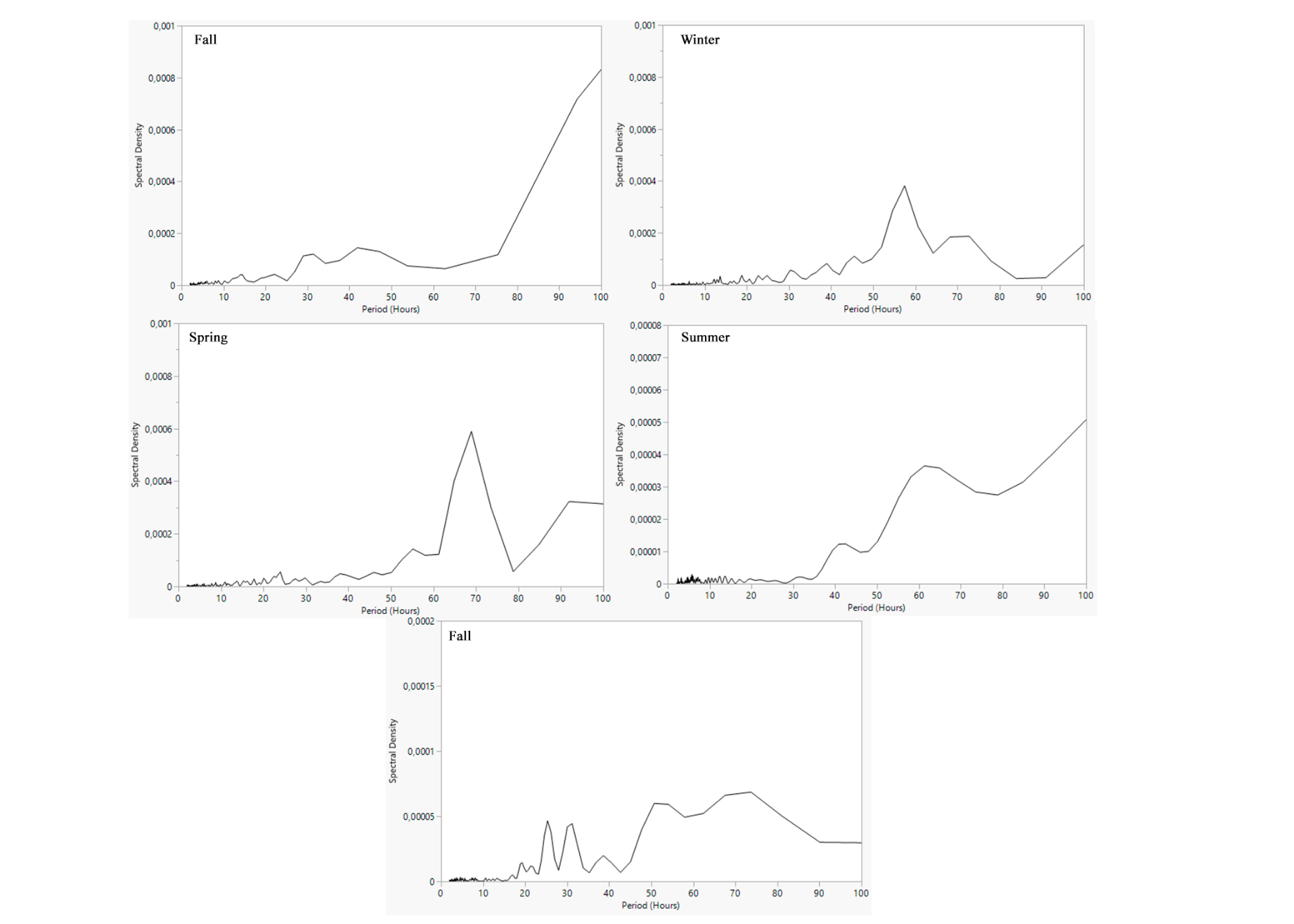


Figure S13: Seasonal spectral density analysis of temperature for the deep zone of the Viento Cave (Canary Islands).
